# Research Note: Validation of a novel scoring system for toe pecking damage in laying hens^☆^

**DOI:** 10.1016/j.psj.2026.107057

**Published:** 2026-05-01

**Authors:** Annemarie J.W. Mens, Malou van der Sluis

**Affiliations:** a Animal Nutrition, Wageningen University & Research 6700 AH Wageningen, the Netherlands; b Animal Breeding and Genomics, Wageningen University & Research 6700 AH Wageningen, the Netherlands

**Keywords:** Abnormal behavior, Damaging behavior, Welfare, Chickens

## Abstract

Toe pecking is an injurious behavior in commercial laying hens, that can result in pain, tissue damage, loss of toe digits, and mortality. However, little is known about the onset, risk factors and potential interventions for toe pecking behavior. To allow systematic and consistent recording of the prevalence and severity of toe pecking wounds in both research and practice, a validated and easily applicable scoring system would be helpful. Here, a scoring system for toe wounds was developed, ranging from a score of 0 (i.e., normal, greasy, no abnormal scales, no wounds, all nails) to a score of 5 (amputation or severe wound with visible bone or tendons). To validate this scoring system, its associated inter- and intra-rater reliability were determined. A total of 50 severed legs collected from carcasses were scored by five independent raters. Per leg, a score was assigned for the 1) inner toe, 2) middle toe, 3) outer toe, 4) hind toe, 5) topside of the foot, and 6) the lower part of the leg, resulting in a set of six scores per leg (300 scores per rater in total). Moreover, a subset of ten legs was scored twice (independently) to assess repeatability. After exclusion of one rater who appeared to have inadvertently mis-scored left versus right feet, a good inter-rater reliability was observed (intraclass correlation coefficient [ICC] = 0.805, 95%-CI = 0.76-0.84, *p* < 0.001). Intra-rater reliability of the four raters was good to excellent, with ICC scores ranging from 0.78 to 0.93. Overall, the scoring system for toe wounds developed in this study can help to systematically and consistently record the prevalence and severity of toe pecking wounds in research and practice.

## Introduction

Toe pecking is an injurious behavior in commercial laying hens (Krause et al., 2011) that can result in pain, tissue damage, loss of toe digits, and mortality (Buitenhuis et al., 2003; Schreiter et al., 2020a). Once injuries are present, toe pecking may escalate, as damaged toes appear to attract further pecking by conspecifics (Buitenhuis et al., 2003). Affected birds may attempt to escape or freeze when receiving toe pecks (Buitenhuis et al., 2003). In addition to direct physical damage, toe pecking has been associated with reduced growth rates (Glatz and Bourke, 2006) as well as psychological and behavioral consequences (Krause et al., 2011).

Despite these welfare implications, toe pecking remains a secondary objective in research, resulting in limited information on its prevalence, causation, and risk factors. Most injurious pecking research has focused on feather pecking, with approximately seventeen times more studies available on feather pecking than on toe pecking (Gebhardt-Henrich et al., 2023). When toe pecking is reported, it is often included as a secondary or ancillary parameter rather than as the primary focus of investigation. Consequently, substantial knowledge gaps remain, including regarding the onset of the behavior itself. While toe pecking is generally described as being directed towards conspecifics (e.g., Buitenhuis et al., 2003; Krause et al., 2011), birds may also peck at their own toes (personal observation).

Toe pecking is likely a multifactorial phenomenon, with several interacting risk factors suggested in literature. These factors include housing or management factors, such as metal slats, high-frequency light sources, a history of *Escherichia coli* infection on the farm within the previous five years, and lesions caused by housing equipment, animal based factors such as dry or scaly skin, early and high egg production, low feed intake at the onset of lay, prior occurrence in pullets, and a generally nervous flock, and environmental factors such as weather changes and direct sunlight, and high mite burdens (all based on a survey among Swiss egg producers) (Gebhardt-Henrich et al., 2023). Other studies have mentioned risk factors such as heat stress, hunger, high light intensity (Glatz and Bourke, 2006) and the hens’ fat metabolism (Trouw Nutrition, 2024). Moreover, a potential genetic component has been suggested, as toe pecking and toe pecking-related mortality appear to occur predominantly in white layer strains (Gebhardt-Henrich et al., 2023; Schreiter et al., 2020b).

To allow systematic and consistent recording of the prevalence and severity of toe pecking wounds in both research and practice, a validated and easily applicable scoring system would be helpful. Existing studies often rely on descriptive or binary measures, which hampers comparison across studies, early detection of outbreaks, and systematic evaluation of risk factors and interventions. Therefore, this study aimed to develop and validate an easy-to-use scoring system for assessing toe damage in laying hens, suitable for both research and commercial circumstances. By providing a validated tool to quantify toe damage severity, this study seeks to facilitate consistent future data collection, improve comparability between studies, and support further research into the prevalence, causes, and prevention of toe pecking in laying hens.

## Materials and methods

### Ethical statement

No animals were culled for the purpose of this study, only legs collected from carcasses at farms were used.

### Development of the scoring system

**Fig. 1:**
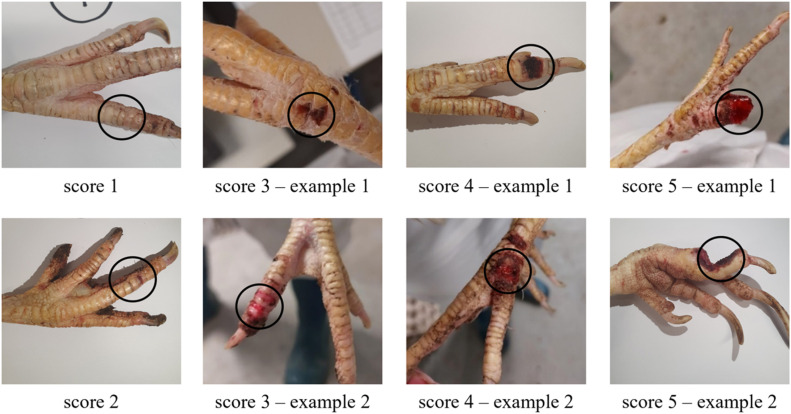
Visual examples of the toe damage scores. The wounds corresponding with the scores are circled.

The development of the scoring system consisted of two steps. Initially, a first version of the scoring system was developed, after which several refinements were made to create the final version of the scoring system. The initial toe scoring system was derived from a (not publicly available) scoring system used by Dutch poultry veterinarians in practice and was adapted for use in an injurious pecking experiment conducted under controlled research conditions. In that experiment, toe pecking damage was scored on a scale from 0 to 5 according to the following criteria: 0 = normal, greasy, no abnormal scales, no wounds, all nails; 1 = dry, flaky with irregular scales; 2 = dry, flaky, or greasy with irregular scales and one red or damaged scale or loss of only the nail; 3 = dry scaly or greasy with irregular scales and multiple small wounds in various locations, wounds are the size of approximately one scale, difference between damage and wound is the surface, damage is shallow, while a wound has more depth, of > 1 mm; 4 = dry scaly or greasy shiny with irregular scales and one or more severe wounds, wounds bigger than the size of approximately one scale and depth of > 2 mm; 5 = amputation of (part of) the toe, this includes severe and deep wounds in which the bone might be visible. As this system was newly adapted, validation was required to evaluate its repeatability and sensitivity. The scoring system was evaluated by seven observers. Independent scoring of wounds and subsequent group discussion indicated that observer scoring was generally consistent within observers, but that the overall assessment across observers retained a degree of subjectivity. Therefore, the toe scoring system was refined to provide clearer definitions and improved differentiation between score categories. In the final version of the toe scoring system, the score descriptions have been made more objective and differences between scores have been clarified. The main difference lies in the further clarification of the size and depth of wounds. The final scoring system is presented in Table 1, in which each of the scores is described. Fig. 1 shows visual examples of the different scores.

**Table 1:** The toe damage scoring system validated in this study.

Score	Description of damage
0	Normal, greasy, no abnormal scales, no wounds, all nails
1	Dry, flaky, no red spots
2	One red spot or wound; approximately 2 mm in size
3	Multiple red spots or wounds; approximately 2 mm in size, surface level
4	At least one wound of approximately >2 mm; deep level. Note: there can be more (smaller) wounds on the toe
5	Amputation or severe wound with visible bone or tendons

### Validation study

For the validation of the scoring system, 50 severed legs collected from carcasses at two farms were used and were kept frozen until the day of scoring. On the day of scoring, each of the legs was assigned a number for identification, by writing down this number on a piece of paper next to each leg. Five persons were asked to independently score all 50 legs using the scoring system from Table 1 in order to assess inter-rater reliability. The inter-rater reliability is an indication for the variation between two or more raters who measure the same group of subjects (Koo and Li, 2016). These five raters had limited experience with investigating toe pecking damage in practice and were not trained to use the tested system. Per leg, a score was assigned for the 1) inner toe, 2) middle toe, 3) outer toe, 4) hind toe, 5) topside of the foot and 6) the lower part of the leg, resulting in a set of six scores per leg (300 scores per rater in total). Subsequently, the observers were asked to repeat the scoring for a randomly selected subset of ten feet (resulting in a total of 60 scores for comparison), without having access to their previous scores for the same legs, to allow for assessment of intra-rater reliability. The intra-rater reliability is an indication of the variation of data measured by one rater across two or more trials (Koo and Li, 2016). The scores from all observers were subsequently combined in a single file for statistical analysis. Inspection of the raw data indicated that one of the raters appeared to have frequently mixed up inner and outer toes (given that separate legs were scored, left and right feet were sometimes difficult to distinguish). The scores for the inner and outer toe from this observer were often opposite to those of the other observers, including instances with a score 5 versus 0 or 1, which are unlikely to indicate different interpretation of wound severity of the same toe. This rater was therefore subsequently excluded from the analyses, resulting in scores of four raters remaining.

### Statistics

R version 4.4.1 was used for the statistical analyses. To assess inter-rater agreement, intraclass correlation coefficients (ICC) were determined using the irr package (Gamer et al., 2019). To test for agreement between the raters, a two-way random effects model was used, in which subjects and raters were considered as randomly chosen from a bigger pool (Gamer et al., 2019). The unit of analysis was set to ‘single’, given the aim to – in future applications of the scoring system – use a measurement from a single rater as the basis of the measurement (Koo and Li, 2016). To determine intra-rater reliability, ICCs were determined per rater using the psych package (Revelle, 2024), using a two-way fixed effects model to test for agreement, with the unit of analysis again set to single. The guidelines of Koo and Li (2016) were adhered to for interpretation of the ICC values, with < 0.5 indicating poor reliability, 0.5-0.75 indicating moderate reliability, 0.75-0.9 indicating good reliability, and > 0.90 indicating excellent reliability.

## Results and discussion

A good inter-rater reliability was observed for the scoring system (ICC = 0.80, 95%-CI = 0.76-0.84, *p* < 0.001). For completeness, the inter-rater reliability was 0.60 when the rater that appeared to have mixed up left and right feet was not excluded (ICC = 0.60, 95%-CI = 0.55 – 0.65, *p* < 0.001). Intra-rater reliability of the four raters was good to excellent, with ICC scores ranging from 0.78 to 0.93 (rater 1 = 0.93 (95%-CI = 0.89-0.96, *p* < 0.001); rater 2 = 0.91 (95%-CI = 0.86-0.95, *p* < 0.001); rater 3 = 0.80 (95%-CI = 0.69-0.88, *p* < 0.001); rater 4 = 0.78 (95%-CI = 0.66-0.86, *p* < 0.001)). The intra-rater reliability of the excluded rater was 0.88 (95%-CI = 0.80 – 0.92, *p* < 0.001).

This good inter-rater reliability score suggests that people that independently score toe wounds using this system will reach similar conclusions regarding the severity of damage. Given that the raters in this study did not receive any training with this scoring system prior to the validation, the scoring system appears to have potential to be used by anyone in practice, regardless of level of experience. It must be noted that one rater was excluded from the analyses, due to apparent mixing up of inner and outer toes (i.e., of left and right legs). This is, however, unlikely to happen when scoring non-severed legs (on live or dead birds), as the distinction between left and right is then straightforward. The good to excellent intra-rater reliability furthermore indicates that raters are consistent in their scoring and will reach similar conclusions independent of the time of scoring.

In this study, previously frozen legs were scored, to obtain a range of toe damage severity and to allow multiple assessors to score the same legs repeatedly without stressing live birds. This freezing and thawing process may, however, have impacted skin coloring and dryness. When scoring live birds in practice, it is expected that both discoloring and dryness of the skin might be even better visible and distinguishable.

Overall, the scoring system for toe wounds developed in this study can help to systematically and consistently record the prevalence and degree of toe pecking wounds in research and practice. Such systematic recording can be the starting point for further investigation of causes of – and interventions for – toe pecking behavior in laying hens.

## CRediT authorship contribution statement

**Annemarie J.W. Mens:** Writing – review & editing, Writing – original draft, Visualization, Validation, Supervision, Project administration, Methodology, Investigation, Funding acquisition, Data curation, Conceptualization. **Malou van der Sluis:** Writing – review & editing, Writing – original draft, Visualization, Validation, Supervision, Project administration, Methodology, Investigation, Funding acquisition, Data curation, Conceptualization.

## Disclosures

The authors declare that they have no known competing financial interests or personal relationships that could have appeared to influence the work reported in this paper.
